# Combining high-resolution cryo-electron microscopy and mutagenesis to develop cowpea mosaic virus for bionanotechnology

**DOI:** 10.1042/BST20160312

**Published:** 2017-11-03

**Authors:** Yulia Meshcheriakova, Alex Durrant, Emma L. Hesketh, Neil A. Ranson, George P. Lomonossoff

**Affiliations:** 1 Department of Biological Chemistry, John Innes Centre, Norwich Research Park, Colney, Norwich NR4 7UH, U.K.; 2 Astbury Centre for Structural Molecular Biology, University of Leeds, Leeds LS2 9JT, U.K.

**Keywords:** cowpea mosaic virus, cryo-electron microscopy, mutagenesis, transient expression, virus-like particle

## Abstract

Particles of cowpea mosaic virus (CPMV) have enjoyed considerable success as nanoparticles. The development of a system for producing empty virus-like particles (eVLPs) of the virus, which are non-infectious and have the potential to be loaded with heterologous material, has increased the number of possible applications for CPMV-based particles. However, for this potential to be realised, it was essential to demonstrate that eVLPs were accurate surrogates for natural virus particles, and this information was provided by high-resolution cryo-EM studies of eVLPs. This demonstration has enabled the approaches developed for the production of modified particles developed with natural CPMV particles to be applied to eVLPs. Furthermore, a combination of cryo-EM and mutagenic studies allowed the development of particles which are permeable but which could still assemble efficiently. These particles were shown to be loadable with cobalt, indicating that they can, indeed, be used as nano-containers.

## Introduction

Cowpea mosaic virus (CPMV) has a long history of use in biotechnology and nanotechnology [1]. All or parts of its RNA genome have been used to create systems for the expression of proteins in plants and its particles have been developed for such applications as epitope display and bioimaging [2–4]. The particles, which consist of 60 copies each of the large (L) and small (S) coat proteins produced via processing of a common precursor (VP60), are particularly suited to these applications as they are highly regular, very robust and can be modified both genetically and chemically. Furthermore, they have been shown to be biocompatible and therefore suitable for *in vivo* applications [5,6].

**Figure 1.**
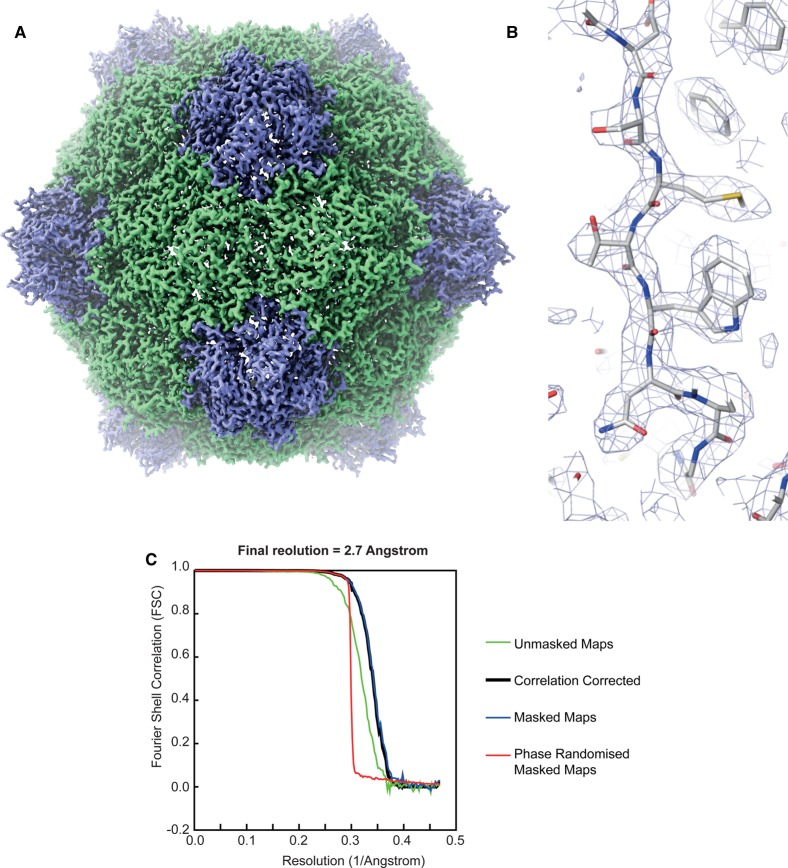
Cryo-EM structure of CPMV eVLP at 2.7 Å resolution. (**a**) EM density map of CPMV eVLP determined by cryo-EM to a global resolution of 2.7 Å. (EMD-3952). The large (L) subunit is displayed in green and the small (S) subunit in blue. Five S subunits interact to form turrets at the five-fold axis. (**b**) Example electron microscopy (EM) density from the cryo-EM reconstruction. (**c**) Fourier Shell correlation (FSC) of masked map, unmasked map, corrected map and phase-randomised map. The resolution reported here was according to the 0.143 criterion. Images produced using ChimeraX UCSF (Goddard et al. [27]), Pymol (Schrodinger [28]) and RELION2.0 (Kimanium et al. [29]).

Traditionally, CPMV particles for biotechnology and nanotechnology were produced through infecting plants with either wild-type (WT) or mutant versions of the virus. While this gives high yields of particles, the majority of these (>90%) contain one or other of the two genomic RNAs (RNA-1, 6 kb; RNA-2, 3.5 kb), with only a small proportion being natural empty particles (top component). This can be a disadvantage since preparations retain infectivity unless specific inactivation steps are taken, and there is limited capacity to load the particles with heterologous material. To address this issue, transient expression has been used to co-express VP60 and the RNA-1-encoded 24K protease necessary for its processing. This results in the formation of particles, termed empty virus-like particles (eVLPs), that are devoid of RNA [7]. Both cryo-electron microscopy (cryo-EM) and crystallographic analysis [8,9] showed that the bulk of the particle structure was identical with that of virus particles produced by infection [8,10,11]; indeed, a cryo-EM reconstruction of eVLPs has recently been produced at a global resolution of 2.7 Å (Figure 1). The only significant difference between the structures of eVLPs and natural virus particles occurs at the C-terminus of S protein where additional amino acids can be resolved in the case of eVLPs; this is a region of 24 amino acids that is often cleaved from mature particles. Overall, these results suggested that eVLPs could be effective substitutes for natural virus particles with the added advantage that they can potentially be loaded. The sequence of VP60 can be readily genetically manipulated and mutant eVLPs can be produced through the co-infiltration of *Nicotiana benthamiana* with separate plasmids containing VP60 and the 24K protease or a plasmid containing both sequences [12,13]. The purpose of this review is to assess progress in the use of eVLPs for a variety of applications.

## Peptide display on the surface of eVLPs

One of the early applications of CPMV particles produced via infection was epitope display [14,15]. This technology involved inserting sequences into exposed loops on the particle surface, the most commonly used one being the βB–βC loop of the S coat protein, though it has proved possible to display peptides at other sites within both the L and S proteins [16,17]. The chimaeras were shown to be able to elicit the production of antibodies against the inserted sequence in a range of experimental animals and, in the case of a chimaera expressing an epitope from Mink enteritis virus, to be able to confer protective immunity in target animals [18]. Crystallographic analysis of the structure adopted by an epitope from human rhinovirus when presented at different positions on the CPMV surface also enabled correlations to be made between the mode of presentation and the antibody response elicited [19,20].

Despite these successes, chimaeras based on infectious CPMV suffer from several disadvantages. The modified particles could potentially spread in the environment, there were limits to the nature of the sequences that could be expressed on the particle surface before infectivity was lost [21] and there was tendency of the insert to be cleaved at its C-terminus [22]. In particular, there was a tendency of chimaeric particles to form large aggregates within infected cells, and it proved difficult or impossible to propagate and purify chimaeras containing inserts with a high pI [21] unless the overall positive charge on the particle was reduced by the inclusion of acidic amino acids [12]. Clearly, the lack of genomic RNA within eVLPs addresses the issue of infectivity, but it was of considerable interest to determine if the use of eVLPs for display could provide a solution to the other issues as well. Therefore, a series of chimaeras was created in which a range of peptides with variable length and charge were inserted into the βB–βC loop of the S coat protein and the yield of the resultant eVLP-based chimaeras assessed (Table 1). The results showed that the yield of chimaeras varied greatly depending on the nature of the peptide inserted, with certain chimaeras giving yields comparable to unmodified eVLPs (0.2–0.3 g/kg leaf wet weight) and others giving barely detectable numbers of particles (<0.01 g/kg). These findings indicate that the use of eVLPs as opposed to infectious virus does not overcome variability of yields found previously [21]. Analysis of the proteins contained within the modified eVLPs suggested that cleavage at the C-terminus of the insert occurs less extensively than had previously been observed with virus particles, with the majority of eVLP-based particles being isolated with an uncleaved loop. The most likely explanation for this is the difference in host used for propagation of eVLPs (*N. benthamiana*) and infectious virus (*Vigna unguiculata*) as cleavage of the C-terminus of the S protein also appears to be more rapid in the latter host.

**Table 1:** Properties of eVLP-based chimaeras Short RGD-containing integrin-targeting peptides and non-RGD tumour-targeting peptides of differing lengths and pI were inserted into the βB–βC loop of S coat protein. Where present, the RGD sequence is shown in bold. The effect of the insert on the yield and assembly or recombinant particles was assessed allowing the chimaeras to be divided into two categories.

High yield/good assembly	Poor yield/assembly problems
TSYN**RGD**STFESK (Fibrinogen α-chain) pI 5.73	GSFG**RGD**SDEWTF λ-receptor on *Escherichia coli* pI 4.03
GVGG**RGD**SGRPIM (Sindbis virus coat protein) pI 9.6	ACMG**RGD**SGGSWI α-lytic protease pI 5.87
VNTANST (tumour-targeting mini-peptide) pI 5.49	LTVSPWY (tumour-targeting mini-peptide) pI 5.52
VPNL**RGD**LQVLAQ (FMDV) pI 5.81	LFHLFIYI (tumour-targeting peptide) pI 6.3
**RGD** pI 5.84	SVVYGRL pI 8.46
NGR pI 9.75	SVSVGMKPSPRP pI 11.0
LDVP (integrin-binding ligand) pI 3.8	CDC**RGD**CFC pI 4.2
G**RGD**NP (integrin-binding site) pI 5.84	YPHYSLPGSSTL pI 6.74

## Modifying the permeability of eVLPs

Given the fact that they contain no encapsidated RNA, it was anticipated that it should be straightforward to load eVLPs with foreign material, especially since natural empty particles produced via infection (top component) had been successfully loaded with metal and metal oxide [23]. Surprisingly, eVLPs were refractory to loading with metals unless first treated with chymotrypsin to remove the C-terminal residues of the S protein [24,25], though some limited permeability to dye molecules could be observed, probably as a result of partial cleavage of the C-terminus of the S protein [26]. Attempts to delete this entire region genetically to obviate the need for enzyme treatment drastically reduced particle formation [8,24]; sequential deletion of parts of the sequence from the C-terminus either allowed the formation of particles that were impenetrable to negative staining or abolished particle assembly (Table 2). Analysis of the cryo-EM structure revealed that the portion of the C-terminal sequence proximal to the main part of the particle makes inter-subunit interactions that play a crucial role in the early stages of particle formation [8] explaining why deletion of the entire 24 amino acids abrogates particle formation.

**Table 2:** Characteristics of deletion and substitution mutants in the 24 amino acid cleavable C-terminus of S coat protein of CPMV eVLPs (cleavage after L189 is indicated by /) The effect of the mutations on particle yield and permeability are shown with WT, indicating that the yield and/or permeability of the particles was similar to that of particles with an intact C-terminus (WT). Permeability was assessed by the ability of the negative stain to penetrate the particles. Abbreviations: N/D: not determined due to the low yield of particles. L189G is shown in bold as it gives a WT yield of permeable particles.

Mutant	Yield	Permeability	Reference
**WT** L/LKFRFRDIERSKRSVMVGHTATAA	WT	WT	[8]
**C-termΔ7** L/LKFRFRDIERSKRSVMV	WT	WT	[8]
**C-termΔ11** L/LKFRFRDIERSKR	WT	WT	[8]
**C-termΔ14** L/LKFRFRDIER	Very low	N/D	[8]
**C-termΔ16** L/LKFRFRDI	Very low	N/D	[8]
**C-termΔ24** L/	Very low	N/D	[8]
ΔL189–190	Very low	Enhanced	This report
ΔL189	WT	WT	This report
L189I	WT	WT	This report
**L189G**	**WT**	**Enhanced**	This report
L189G/L190G	Very low	N/D	This report
L189F/L190F	Moderate	WT	This report

**Figure 2.**
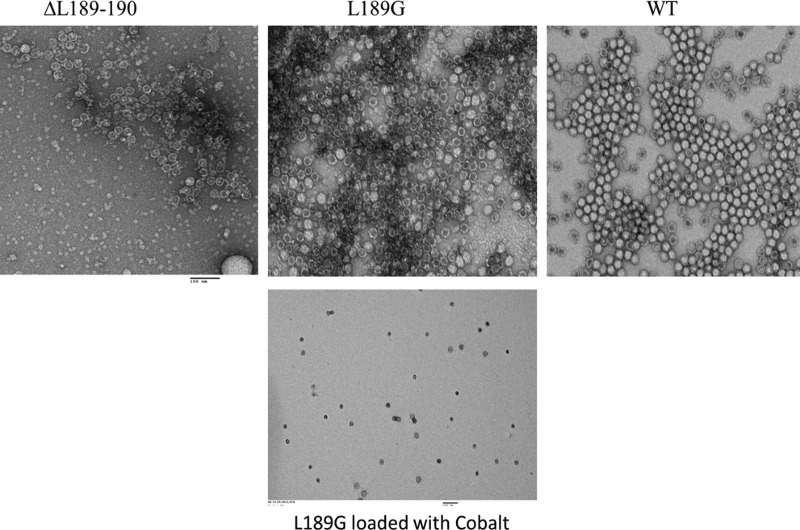
TEM of enhanced permeability mutants ΔL189-190 and L189G and WT eVLPs. The permeability of the particles assessed by the extent of penetration of the 2% (w/v) uranyl acetate negative stain (upper images) and loading of L189G particles with cobalt (lower image).

In an attempt to produce eVLPs that can assemble yet are permeable, a series of additional mutants were created, concentrating on the region where cleavage occurs (between L189 and L190 of the S protein; Table 2). As with the deletion mutants, most of the mutations either abolished assembly or led to the formation of particles which were as impermeable as wild-type eVLPs. The exception was mutant L189G in which the last amino acid visible in the cleaved form of the S protein was mutated to glycine. This mutant gave wild-type levels of particles which appeared to stain-penetrable (Figure 2). To confirm the permeability of the L189G mutant particles, they were subjected to the cobalt-loading protocol previously applied successfully to CPMV top component and chymotrypsin-treated eVLPs [23,24]. Transmission electron microscopy in the absence of negative stain revealed that cobalt could be successfully deposited within the L189G particles, indicating that such mutations could be a route to making permeable, and therefore loadable, eVLPs, obviating the need for protease digestion.

**Figure 3.**
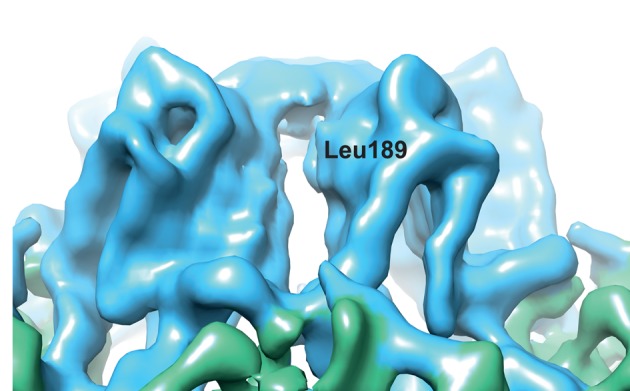
Cryo-EM reconstruction of eVLP, with a Gaussian filter to 2 Å applied. A zoomed-in view of a CPMV turret (made up of five S subunits) showing a pore allowing access to the interior of CPMV capsid. This pore is usually occluded by the C-terminal of S subunit. We propose that the flexibility of this region is increased with the L189G mutation. The approximate position of Leucine 189 in the eVLP is indicated.

The exact position of L189 in the eVLP structure is unknown, as residues 184–189 are not visible in the atomic model derived from the cryo-EM reconstruction [8]. However, the approximate position can be deduced as L190 is visible and the EM density for the amino acid backbone can be visualised. We predict that the hydrophobic leucine side chain is usually buried in the protein structure and therefore shielded from the external environment, stabilising the structure of the S subunit C-terminus. We predict that the L189G mutation weakens this hydrophobic interaction and increases the flexibility of the whole C-terminus. This increased flexibility reveals a large pore allowing access to the interior of the capsid (Figure 3). Despite increasing C-terminal flexibility and allowing access to the interior of particle, the L189G substitution does not abolish the C-terminal interactions necessary for particle formation [8]. The ability to produce high levels of permeable capsids without the need for enzymatic treatment should promote the take up of eVLPs as nano-containers.

## Conclusions

The availability of a non-infectious form of CPMV particles (eVLPs) has opened new possibilities for the use of such particles in biotechnology and nanotechnology. However, a vital prerequisite for such new developments was the demonstration, by cryo-EM, that eVLPs are accurate structural mimics of natural CPMV particles. Such studies also informed the development of permeable capsids for loading purposes. Future studies may also make use of cryo-EM technology to analyse the structures of derivatised versions of eVLPs, once more underlining the benefit of combined genetic and structural studies.

## Database depositions

Cryo-EM reconstruction of CPMV eVLP is deposited in the EM Data Bank under accession code EMD-3952.

## Abbreviations

CPMV: cowpea mosaic virus
cryo-EM: cryo-electron microscopy
eVLPs: empty virus-like particles
WT: wild type
